# Emotion Recognition Pattern in Adolescent Boys with Attention-Deficit/Hyperactivity Disorder

**DOI:** 10.1155/2014/761340

**Published:** 2014-07-08

**Authors:** Nikoletta Aspan, Csilla Bozsik, Julia Gadoros, Peter Nagy, Judit Inantsy-Pap, Peter Vida, Jozsef Halasz

**Affiliations:** ^1^Vadaskert Child Psychiatry Hospital, 5 Lipotmezei Street, Budapest 1021, Hungary; ^2^School of Ph.D. Studies, Semmelweis University, 26 Ulloi Street, Budapest 1085, Hungary; ^3^Institute of Psychology, University of Debrecen, 1 University Square, Debrecen 4010, Hungary; ^4^Alba Regia University Centre, Obuda University, 45 Budai Street, Szekesfehervar 8000, Hungary

## Abstract

*Background*. Social and emotional deficits were recently considered as inherent features of individuals with attention-deficit hyperactivity disorder (ADHD), but only sporadic literature data exist on emotion recognition in adolescents with ADHD. The aim of the present study was to establish emotion recognition profile in adolescent boys with ADHD in comparison with control adolescents. *Methods*. Forty-four adolescent boys (13–16 years) participated in the study after informed consent; 22 boys had a clinical diagnosis of ADHD, while data were also assessed from 22 adolescent control boys matched for age and Raven IQ. Parent- and self-reported behavioral characteristics were assessed by the means of the Strengths and Difficulties Questionnaire. The recognition of six basic emotions was evaluated by the “Facial Expressions of Emotion-Stimuli and Tests.” *Results*. Compared to controls, adolescents with ADHD were more sensitive in the recognition of disgust and, worse in the recognition of fear and showed a tendency for impaired recognition of sadness. Hyperactivity measures showed an inverse correlation with fear recognition. *Conclusion*. Our data suggest that adolescent boys with ADHD have alterations in the recognition of specific emotions.

## 1. Introduction

According to the data of two major meta-analyses, attention deficit/hyperactivity disorder (ADHD) is the most common neurodevelopmental disorder, with a worldwide prevalence rate of 5–7 percent [1, 2]. The core symptoms of the disorder are inattention, hyperactivity, and impulsivity [3], but children and adolescents with ADHD might have multiple comorbid psychiatric conditions including externalizing and internalizing disorders [4]. There are still many open issues in the useful interventions and the optimal treatment of affected individuals. A growing but still sporadic literature data highlighted that, in addition to the core symptoms, altered social information processing might be responsible for certain aspects of social dysfunction in patients with ADHD. Data from the last decade suggest that impaired social skills and behavioral problems in children and adolescents with ADHD were not fully explained by additional comorbidities or secondary consequences of disturbed executive functions [5, 6]. Understanding possible deficits in emotion recognition might be crucial in the development of proper interventions targeting behavioral problems in children and adolescents with ADHD.

Facial affect recognition is a major component of social behavior and might be partly responsible for social dysfunction described in children and adolescents with ADHD [5]. Singh et al. reported detailed emotion recognition pattern of children with ADHD between 5 and 13 years [7]. In this particular study, the authors did not study unaffected children but used emotion recognition data from other studies. Later reports with control subjects confirmed an impaired emotion recognition profile in children with ADHD compared to controls [8–16]. In the above studies, the children with ADHD were mainly under 13 years [7, 8, 10–14, 16], albeit with different methods, a rather general pattern of impaired emotion recognition was described.

Less information on emotion recognition is available in adolescents and adults with ADHD. To our best knowledge, there is no literature data on the emotion recognition profile in adolescents with ADHD. In two studies, some adolescents above 13 years were also included, but the mean age of the participants was between 8 and 10 years [9, 15]. In two additional studies, children and adolescents between 8 and 17 years [17] and between 6 and 18 years [18] were the study subjects. In these latter studies, no separate data on adolescents were reported. In children and adolescents with ADHD (8–17 years), Williams et al. described deficits in the recognition of anger and fear [17]. In adults with ADHD, the intensity of experienced emotions moderated affect recognition; experienced emotions were inversely related to affect recognition [19]. Compared to controls, Miller et al. described more errors in the recognition of expressions related to fear in adults with ADHD [20].

The effect of comorbidities was also studied on facial emotion recognition patterns in children with ADHD. In children, between 7 and 13 years, ADHD with or without conduct problems was accompanied by decreased affect recognition, and the ADHD group made errors in random order [16]. In a study of Sinzig et al., children with ADHD, autism and ADHD and autism without ADHD were studied (between ages of 6 and 18 years), and an independent effect of ADHD on overall emotion recognition was described [18]. Thus, it seems that ADHD has an independent effect on facial affect recognition.

According to population studies, facial affect recognition keeps developing until late adolescence [21]. ADHD seems to modify emotion recognition profile in children [7–14, 16], in mixed groups of children and adolescents [15, 17, 18] and in adults [19, 20] as well, albeit the pattern seems to shift with age from a general difficulty in children to rather specific alterations in adults. A rather specific alteration might suggest that the effect cannot be solely attributed to attention problems but might be related to specific alterations in social cognition [5, 6] and might lead to specific therapeutic interventions. Our study targeted adolescents with ADHD directly and a link between emotion recognition and ADHD related behavioral parameters was suggested.

The aims of the present study were (i) to establish an emotion recognition profile in adolescent boys with ADHD in comparison with control adolescents and (ii) to assess the relationship between hyperactivity/inattention problems and the recognition of emotions in the present sample.

## 2. Methods

### 2.1. Subjects

Adolescent boys aged between 13 and 16 years (14.7 ± 0.1, mean ± SEM) participated in the study after giving informed consent. According to the ethical standards in Hungary, both the adolescents and their parents gave their written consent to the experimental procedure. The study has been supervised by the Joint Ethical Committee of Szent Imre/Szent Janos Hospitals and the General Psychology Research Ethical Committee in Hungary. Eighty percent of interviewed individuals agreed to participate in the study. The clinical data collection was performed at Vadaskert Child Psychiatry Hospital, while data were also collected from control adolescents with matched IQ, age, geographical location, and family background.

The clinical adolescent sample was randomly selected from an inpatient behavioral treatment unit of the hospital, where a 5-day behavioral therapy program is provided for adolescents with ADHD. This inpatient intervention was only provided to motivated individuals; thus individuals with severe conduct problems did not take part in these groups. The 5-day long inpatient program provided a cognitive-behavioral therapeutic intervention, and among the inclusion criteria for adolescents with ADHD was the motivation instead of the severity of the condition. The study was performed on the first or second day of the behavioral therapy. The clinical sample consisted of adolescent boys with ADHD between 13 and 16 years, where the diagnosis was formulated by an experienced child psychiatrist, then confirmed by an independent child psychiatrist (inclusion criteria). The diagnosis of ADHD was established using DSM-IV criteria (the subjects also fulfilled ICD-10 criteria); all adolescents were diagnosed with the combined type of ADHD. The exclusion criteria were (i) autism spectrum disorder, (ii) previous diagnosis of psychotic disorder, (iii) substance use in the last month, and (iv) Raven IQ performance below 80. In the clinical sample, data from 22 individuals with ADHD were used, 2 from the 24 patients had to be excluded (one with autism spectrum disorder; one with Raven IQ performance was below 80). The following ICD-10 comorbid clinical diagnoses were also present: childhood emotional disorder (*N* = 6, F93.80), conduct disorder, unspecified or mixed disorder of conduct and emotions (*N* = 3, F91.90, F92.90), obsessive-compulsive disorder (*N* = 2, F42.20), and Tourette syndrome (*N* = 1, F95.20).

Past psychotropic medication was not among the exclusion criteria in the clinical group. Documented previous (any time) psychotropic medication was present in 11 cases. The most frequently used previous medication was methylphenidate (10 cases) and in 1 case imipramine. In the case of methylphenidate, adolescents were medication-free for at least 72 hours before the emotion recognition task. Within the clinical group, previous psychotherapy was present in 14 cases. In the case of controls, the following additional exclusion criteria were used: (i) earlier use of psychotropic medications, (ii) earlier psychological or psychiatric treatment, and (iii) parental score on hyperactivity (Strength and Difficulties Questionnaire) was >6. In the case of controls, two boys fulfilled the above criteria (high hyperactivity score), and the data of 22 adolescents were used.

### 2.2. Procedure

After informed consent, adolescents participated in a test of emotion recognition and their performance in the Raven's Progressive Matrices was also assessed. Behavioral data were obtained both from parents and the adolescents by the means of the Strengths and Difficulties Questionnaire and the Inventory of Callous-Unemotional Traits. In addition to the clinical interview and diagnosis formulation, the procedure lasted about 60–70 minutes for the participants.

### 2.3. Instruments

#### 2.3.1. Strengths and Difficulties Questionnaire (SDQ)

The SDQ is used as a general screening questionnaire on the behavioral problems of children and adolescents [22–25]. A Hungarian translation is also available (parent- and self-report); population and clinical standards have also been established [26–28]. Both the parent- and self-report versions of the SDQ contain 25 items, 5 questions in each of the 5 scales (Emotional Symptoms Scale, Conduct Problems Scale, Hyperactivity Scale, Peer Problems Scale, and Prosocial Scale). The Hyperactivity Scale contains items for hyperactivity, inattention, and impulsivity. The maximum is 10 points for each scale. Higher sores represent more severe behavioral problems, except in the case of the Prosocial Scale, where higher scores mean better performance (strengths).

#### 2.3.2. The Inventory of Callous Unemotional Traits (ICU)

The ICU was used to control the effect of psychopathic traits on emotion recognition, as in earlier studies children with psychopathic tendencies showed marked impairment in facial affect recognition [29]. Children and adolescents with ADHD have major vulnerability towards externalization problems [4] and also to callous-unemotional traits [30]. The ICU was introduced by Frick and coworkers, and for an extensive review on callous-unemotional traits (e.g., lack of guilt, absence of empathy, and callous use of others) see [31]. The ICU has 24 items; both parent-reported and self-reported versions are available, and the Hungarian version was also introduced [32]. In the present study, the overall ICU score was used. The maximum ICU score is 72 points; the higher numbers represent higher levels of callous-unemotional traits [31].

#### 2.3.3. Facial Expressions of Emotion-Stimuli and Tests (FEEST)

Based on the six universal basic emotions described by Ekman and colleagues [33], the computerized and extended version of the original 60 faces test, FEEST, was used [34]. The “emotion” test was used in the study. During the procedure, subjects choose a label for the emotional content (anger, disgust, fear, happiness, sadness, and surprise) of the faces visible on the screen. The maximum number of correct responses for each emotion is 10, with a maximum total score of 60. The images are displayed in a random order; the stability of responses were also evaluated in six blocks including ten images each.

#### 2.3.4. Raven Test of Progressive Matrices

Standard progressive matrices were used [35]. The 60 progressive steps were standardized according to the Hungarian gender and age, and a general IQ measure was outlined. The Raven performance scores were between 80 and 137.

### 2.4. Statistical Analysis

The analysis was performed with the STATISTICA 7.0 software package. Behavioral data (SDQ) were analyzed by repeated measure ANOVA (factor 1: group, repeated variable: information source (parent- and self-report)). Distinct components of emotion recognition were analyzed by general linear model, where age and Raven IQ were used as covariates. Repeated measure ANOVA was also used to assess progress-related differences in overall emotion recognition, where the correct responses were analyzed in blocks of ten images. Fisher* post hoc* comparison was performed if it was appropriate. Spearman correlations were used to assess the relationship between behavioral variables and recognition of emotions. Partial correlations were also run to test the independent effect of behavioral variables. The level of significance was set at *P* < 0.05.

## 3. Results

### 3.1. General and Behavioral Data (SDQ, ICU)

The age (mean ± SEM; control: 14.5 ± 0.2; ADHD: 14.2 ± 0.2 years; *F*_(1,42)_ = 2.350, *P* < 0.133) and Raven performance (mean ± SEM; control: 110.2 ± 2.3; ADHD: 108.7 ± 3.7; *F*_(1,42)_ = 0.127, *P* < 0.724) were not different between the two groups.

The parent-reported scores of Conduct Problems Scale and Hyperactivity Scale (SDQ) were significantly higher than self-reported scores. A prominent group effect of ADHD was present in all scales except the Prosocial Scale (Table 1). The highest group effect could be observed in the scores of the Hyperactivity Scale.

The parent-reported ICU score was significantly higher than self-reported score of ICU, and a prominent group effect of ADHD was also present (Table 1).

### 3.2. FEEST Scores

**Figure 1:**
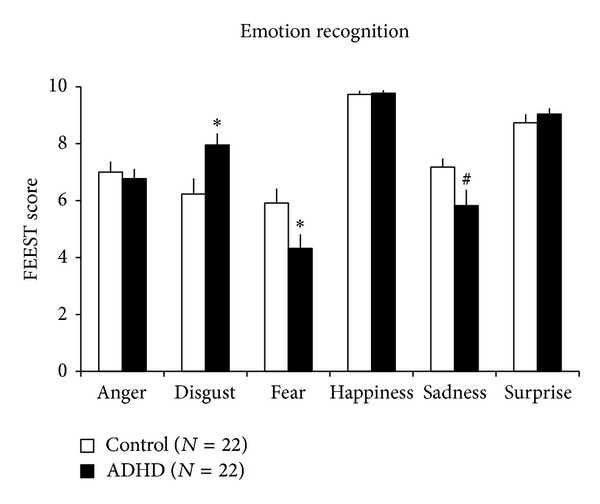
FEEST scores by distinct emotions in the experimental groups. ADHD, adolescent boys (13–16 years) with attention-deficit/hyperactivity disorder; control, control adolescent boys (matched on age and Raven IQ); ∗statistically significant difference (*P* < 0.05) from controls; ^#^tend to differ statistically (*P* < 0.06) from controls. Data are expressed as mean ± SEM.

Recognition of distinct emotions was analyzed by the general linear model (age and Raven IQ were used as covariants). Compared to control adolescents, adolescents with ADHD were more sensitive in the recognition of disgust (*F*_(1,40)_ = 6.228, *P* < 0.017), were significantly worse in the recognition of fear (*F*_(1,40)_ = 4.767, *P* < 0.035), and showed a tendency toward impaired recognition of sadness (*F*_(1,40)_ = 3.771, *P* < 0.056) (Figure 1). The recognition of anger (*F*_(1,40)_ = 0.077, *P* < 0.78), happiness (*F*_(1,40)_ = 0.219, *P* < 0.64), and surprise (*F*_(1,40)_ = 2.044, *P* < 0.16) did not show significant differences between the two groups.

**Figure 2:**
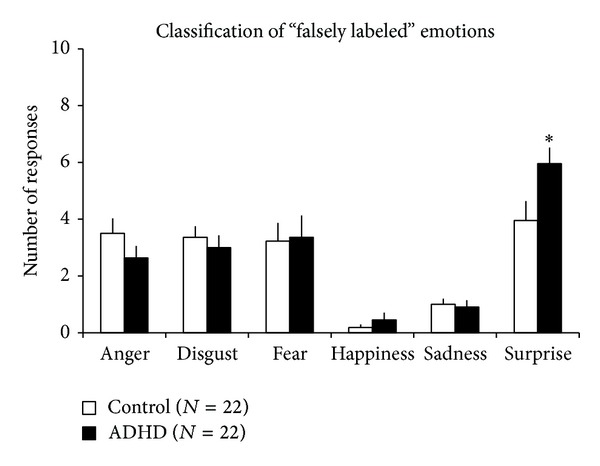
The classification of “falsely labeled” emotions in the experimental groups. ADHD, adolescent boys with attention-deficit/hyperactivity disorder; control, control adolescent boys; ∗statistically significant difference (*P* < 0.05) from controls. Data are expressed as mean ± SEM.

A different analysis was also performed to describe the responses for “falsely labeled” emotions. Compared to the control group, adolescent boys with ADHD classified “falsely labeled” emotions as surprise significantly more often (*F*_(1,40)_ = 5.074, *P* < 0.030) (Figure 2). The summary of the labeling matrices of the two groups is presented in Table 2.

**Figure 3:**
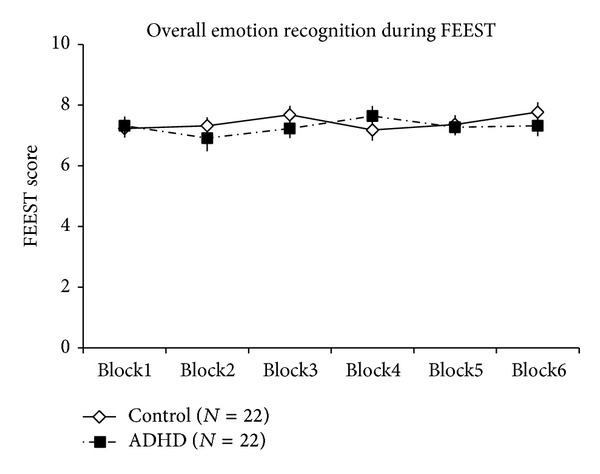
The number of correct responses in six blocks during the emotion recognition test. ADHD, adolescent boys with attention-deficit/hyperactivity disorder; control, control adolescent boys. Data are expressed as mean ± SEM.

Additionally, the overall emotion recognition was also analyzed in six consecutive blocks (10 stimuli in each block). In the overall emotion recognition, no group effect was present (*F*_(1,42)_ = 0.448, *P* < 0.51). Moreover, neither block effect was present (*F*_(5,210)_ = 0.591, *P* < 0.71), nor the interaction between group and block effect was significant (*F*_(5,210)_ = 0.788, *P* < 0.56) (Figure 3).

### 3.3. Spearman Correlations and Partial Correlations between the Recognition of Emotions and Behavioral Variables

As marked differences were present between the two groups in the recognition of disgust, sadness, and fear, Spearman correlations between the variables of emotion recognition and SDQ variables were also run.

The recognition of disgust was positively correlated with parent-reported hyperactivity (Spearman* R* = 0.376, *P* < 0.012) and self-reported conduct scores (Spearman* R* = 0.310, *P* < 0.040). The recognition of sadness did not correlate with SDQ measures. The recognition of fear was inversely correlated with both parent- and self-reported hyperactivity measures (parent-report: Spearman* R* = −0.315, *P* < 0.037; self-report: Spearman* R* = −0.502, *P* < 0.0005); while the recognition of fear showed a tendency for an inverse correlation with parent-reported conduct scores (Spearman* R* = −0.271, *P* < 0.08). The other behavioral scales of SDQ did not show any correlation with the recognition of disgust or fear.

Parent-reported ICU scores showed a tendency for an inverse correlation with the recognition of fear (Spearman* R* = −0.283, *P* < 0.07), and a marked inverse correlation between self-reported ICU scores and the recognition of sadness was present (Spearman* R* = −0.418, *P* < 0.005).

Partial correlation analyses between the behavioral variables (parent-reported and self-reported conduct and hyperactivity scores) and fear recognition were also run. In the recognition of fear (multiple* R* = 0.475,* F* = 2.849, and *P* < 0.04), only the effect of self-reported hyperactivity scores remained significant (*β* = −0.50, *P* < 0.012). The effect remained unaltered after including ICU variables into the analysis (*β* = −0.49, *P* < 0.022).

## 4. Discussion

The main results of the present study were that (i) adolescent boys with ADHD were more sensitive in the recognition of disgust and had worse performance in the recognition of sadness and fear, compared to age- and Raven-IQ-matched controls; (ii) hyperactivity measures were positively correlated with the recognition of disgust and inversely correlated with the recognition of fear in our sample.

### 4.1. Pattern of Facial Affect Recognition in Adolescents with ADHD

In the present study, marked differences were found in the recognition of distinct emotions between the clinical and the control groups. Compared to control boys, adolescent boys with ADHD were more sensitive in the recognition of disgust, and a dysfunction was present in the recognition of fear and data indicated a difficulty in the recognition of sadness.

To our best knowledge, increased sensitivity in the recognition of disgust in subjects with ADHD has not been described earlier in the literature, but a relatively better recognition of disgust (compared to the recognition of other emotions) was indicated in children [11, 17] and adults with ADHD [19]. In contrast to our results, in a study with children between 7 and 12 years, ADHD scores were inversely correlated with disgust [14]. The meaning of increased sensitivity toward disgust in adolescent boys with ADHD is unclear, but together with an impaired recognition of sadness and fear, it might represent a pronounced vulnerability in social contexts.

Impaired recognition of sadness has been reported in children [11, 13] and in adolescents [17], but not in adults with ADHD [19, 20]. Our data show a marginal impairment in the recognition of sadness, indicating a transition-like period between unaltered recognition of sadness in adults and impaired recognition of sadness in children and adolescents. In the study of Williams et al. the age of the subjects was between 8 and 17 years (13.8 in average) [17]; the inclusion of younger subjects than our sample might be responsible for impaired recognition of sadness in that study. Interestingly, only ICU scores showed an inverse correlation with the recognition of sadness in our study.

In contrast to the recognition of sadness, an impaired recognition of fear was consistently reported both in children and adults [13, 17, 19, 20]. Moreover, Boakes et al. described impairment in fear recognition with static and dynamic representations and described an inverse correlation in children between ADHD scores and fear recognition [14]. The impairment in the recognition of fear seems robust and consistently described throughout studies in patients with ADHD. Our data showed inverse correlation between fear recognition and hyperactivity scores. Altogether, the alterations reported in the recognition of fear might have importance in the social dysfunction in adolescents with ADHD.

### 4.2. Possible Mechanisms Underlying Specific Alterations in Emotion Recognition in Individuals with ADHD

#### 4.2.1. Attention Problems

The first possible explanation of altered emotion recognition in subjects with ADHD is the presence of attention problems, as core symptom of ADHD [3]. Visual attention deficit has been suggested as a moderator of emotion recognition in children with ADHD. Shin et al. demonstrated that the visual attention-component is important in the contribution of errors performed by children with ADHD during emotion recognition [15]. Bias in the recognition of emotions in ADHD was also connected to attention problems in other studies as well, especially when the complexity of stimuli was considerably high. For example, da Fonseca suggested that not only children with ADHD have a problem with the recognition of emotions, but also their attention difficulty affects the recognition of emotions on the basis of contextual information [9]. A generally impaired perception of affect from facial stimuli and intonation of speech was concomitantly described in children with ADHD between 7 and 12 years [8], thus not a specific element of social behavior, but a wider origin of social dysfunction was suggested. However, Yuill and Lyon reported higher difficulty in the interpretation of emotional than nonemotional task; thus, according to the interpretation of the authors, the poor performance could not be fully explained by attention shift [13]. Our study did not target the above questions directly. Interestingly, the overall emotion recognition did not change during the test and was not different between adolescent boys with ADHD and controls. Albeit the emotion recognition test was considerably short (15 minutes), we might suggest that at least during this limited period major alterations in the selective attention toward the presented stimuli were unaffected. In our study, subjects had a considerably high Raven IQ performance, which may also modify interpretation. As in our study adolescent boys with ADHD were markedly sensitive in recognizing disgust, and an impaired recognition of fear was present; these data might suggest a rather specific emotion recognition pattern in contrast to a generally higher threshold of emotion recognition due to attention problems.

#### 4.2.2. Comorbid Conditions

A possible second explanation for altered emotion recognition in subjects with ADHD is the presence of comorbid disorders. In this respect, autism spectrum disorder has major importance, where major alterations in emotion recognition (including the recognition of fear) have been described. In a study performed by Sinzig and colleagues, an independent effect of ADHD on overall emotion recognition was described in children with autism [18]. In our study, the presence of autism spectrum disorder was one of the exclusion criteria; thus, a major effect cannot be attributed to the effect of possible autism spectrum disorder in our sample.

Another major comorbid condition is conduct disorder. Interestingly, impaired fear recognition is a marked phenomenon in antisocial personality disorder [36] and throughout antisocial development [29, 37, 38]. As conduct disorder can be considered as one of the most prevalent comorbid conditions of subjects with ADHD [4], interpretation of conduct symptoms has high importance in understanding the present results. Nevertheless, the phenomenon described as “fear blindness” in children with psychopathic traits and conduct disorder has been used as a possible target of therapeutic intervention throughout the early stages of antisocial development [39], and similar intervention might also be of interest in children with ADHD. In children (7–13 years) with ADHD, the presence of conduct disorder did not modify the emotion recognition pattern [16]. In a study with children and adolescents between 7 and 18 years, subjects with ADHD and conduct disorder were grouped together in comparison with bipolar disorder, severe mood dysregulation, mood disorder, and controls [40]. In this sample, no separate effect of ADHD and conduct disorder on emotion recognition was reported. In our sample, parent-reported conduct scores in the clinical group were considerably high, and also high ICU scores were associated with a marginal impairment in the recognition of fear. However, partial correlations revealed an independent effect of hyperactivity scores in our sample. Still, one cannot exclude the role of dysfunctions in overlapping limbic circuits in ADHD and conduct disorder.

#### 4.2.3. Central Mechanisms

At present, the central mechanisms behind impaired fear recognition in subjects with ADHD are unclear. In the study of Brotman et al. left amygdalar hyperactivity was reported in children with ADHD during coding fearfulness on facial stimuli compared to controls [41]. Posner et al. published increased right amygdalar activity to subliminal presentation of fearful faces in adolescents with ADHD compared to controls [42]. In a different experiment, adolescents with ADHD were similar to control subjects in the amygdalar responses on fearful expressions but showed increased reactions compared to youths with callous-unemotional traits [43]. These results are not conclusive in relation to amygdalar hyperactivity and further studies are required to outline the central mechanisms underlying social deficits in general and facial affect recognition in particular in children and adolescents with ADHD. In addition to the role of amygdala, impaired amygdala-prefrontal interplay was suggested behind the social dysfunctions of ADHD [6].

### 4.3. Limitations of the Study

The limitations of the study are (i) the use of static images during emotion recognition and (ii) the use of emotion recognition test originally developed for adults. The study was based on the number of the recognized emotions on static images, while dynamic approaches were also introduced in the methodology of studies dealing with emotion recognition, albeit only in a minority of the studies focusing on ADHD [14]. With dynamic approaches, a better face-validity to real-life situations can be established. In the present study, only a basic well-known process based on static images was applied, and the differences could be interpreted only in this context. Also, the FEEST procedure was originally developed for adults [34]. However, according to literature data, FEEST-like procedures could be reliably applied in studies of adolescents [37, 44].

### 4.4. Conclusions

Our data indicate that adolescent boys with ADHD have a specific emotion recognition pattern, characterized by an increased sensitivity in the recognition of disgust and an impaired recognition of fear, with a tendency for the impaired recognition of sadness. Impaired fear recognition was strongly associated with hyperactivity scores. According to literature data, these alterations might have a major importance in understanding the social deficits in children and adolescents with ADHD.

## Figures and Tables

**Table 1:** Behavioral variables (Strengths and Difficulties Questionnaire, SDQ; Inventory of Callous-Unemotional Traits, ICU).

SDQ	Parent report		Self-report		Group effect *F*_(1,42)_	Group effect *P*<	Report effect *F*_(1,42)_	Report effect *P*<	Interaction effect *F*_(1,42)_	Interaction effect *P*<
	ADHD (*N* = 22)	Control (*N* = 22)	ADHD (*N* = 22)	Control (*N* = 22)						
Emotional	3.0 ± 0.6∗	1.6 ± 0.3	3.8 ± 0.6∗∗	1.6 ± 0.3	12.630	0.001	0.857	ns	0.857	ns
Conduct	5.1 ± 0.6∗∗	1.4 ± 0.3	4.8 ± 0.5∗∗	2.0 ± 0.3	46.430	0.001	0.192	ns	1.726	ns
Hyperactivity	8.7 ± 0.2∗∗	2.7 ± 0.3	6.6 ± 0.5∗∗	3.5 ± 0.4	136.847	0.001	3.404	0.07	19.730	0.001
Peer relations	3.8 ± 0.5∗	2.0 ± 0.3	3.4 ± 0.5∗∗	1.1 ± 0.2	16.442	0.001	6.173	0.02	0.595	ns
Prosocial	5.8 ± 0.4∗	7.2 ± 0.4	6.7 ± 0.4	6.5 ± 0.3	1.612	ns	0.028	ns	9.201	0.01
ICU score	36.9 ± 2.2∗∗	25.4 ± 1.8	30.0 ± 2.3∗∗	23.8 ± 1.7	14.269	0.001	7.396	0.01	2.814	ns

Data are expressed as mean ± SEM. ADHD: adolescent boys with attention-deficit/hyperactivity disorder; control: adolescent boys matched for age and Raven IQ; ∗significantly different (*P* < 0.05) from the respective control in *post hoc *comparison; ∗∗significantly different (*P* < 0.01) from the respective control in *post hoc *comparison. In the case of ICU, only data from 43 subjects were analyzed.

**Table 2:** The classification of emotions in the two experimental groups (overall).

ADHD (Control)	Responses, *n* = 1320					
	Anger	Disgust	Fear	Happiness	Sadness	Surprise
Stimuli						
Anger, *n* = 220	**149 (154)**	27 (25)	14 (11)	1 (1)	9 (12)	20 (17)
Disgust, *n* = 220	40 (69)	**175 (137)**	2 (10)	0 (0)	3 (4)	0 (0)
Fear, *n* = 220	7 (4)	16 (24)	**95 (130)**	3 (0)	5 (1)	94 (61)
Happiness, *n* = 220	1 (0)	0 (0)	0 (2)	**215 (214)**	2 (2)	2 (2)
Sadness, *n* = 220	10 (4)	20 (23)	44 (27)	3 (1)	**128 (158)**	15 (7)
Surprise, *n* = 220	0 (0)	3 (2)	14 (21)	4 (1)	0 (4)	**199 (192)**

Total, *n* = 1320	207 (231)	241 (211)	169 (201)	226 (217)	147 (181)	330 (279)

Data are expressed as sums for the 22 individuals in both groups, ADHD and control. “Stimuli” represent the official categorization of the images, while “responses” represent the subject classification of the displayed images during the facial affect recognition procedure (FEEST). The maximum number of correct images individually was 10 per emotions, altogether 220 for the 22 subjects in each group. Correct responses for the given emotional stimuli were highlighted.
